# AhR-Mediated, Non-Genomic Modulation of IDO1 Function

**DOI:** 10.3389/fimmu.2014.00497

**Published:** 2014-10-15

**Authors:** Maria Teresa Pallotta, Francesca Fallarino, Davide Matino, Antonio Macchiarulo, Ciriana Orabona

**Affiliations:** ^1^Pharmacology Section, Department of Experimental Medicine, University of Perugia, Perugia, Italy; ^2^Department of Pharmaceutical Sciences, University of Perugia, Perugia, Italy

**Keywords:** IDO1, AhR, ITIM, ubiquitin ligase complex, tryptophan metabolism, l-kynurenine, immunoregulation, dendritic cells

## Abstract

The evolutionary process has conferred a dual – enzymatic and signaling – function on the ancestral metabolic enzyme indoleamine 2,3-dioxygenase 1 (IDO1), which has long been known for converting the essential amino acid tryptophan (TRP) into neuroactive and immunoactive catabolites (kynurenines). In addition to TRP catabolic activity, phosphorylated immunoreceptor tyrosine-based inhibitory motifs, present in the IDO1 protein, act as docking sites for different molecular partners, which activate positive (transcriptional) or negative (post-translational) modulation of IDO1 protein. The ligand-operated transcription factor aryl hydrocarbon receptor (AhR) contributes to *Ido1* transcription, and it can be operated by both exogenous and endogenous ligands, including l-kynurenine itself, the first byproduct of TRP catabolism. Ligand-bound AhR is also a component of a ubiquitin ligase complex responsible for regulatory proteolysis of different target proteins. Because IDO1 half-life is controlled by the ubiquitin–proteasome system, we here discuss the possibility that AhR, in addition to enhancing *Ido1* transcription, contributes to IDO1 regulation by a non-genomic mechanism affecting the protein’s half-life.

## Introduction

Fine tuning of immune reactivity is guaranteed by the recruitment of enzymes with disparate and pleiotropic functions. Indoleamine 2,3-dioxygenase 1 (IDO1) was first recognized as an immune regulator in pregnancy (1), and subsequently, in numerous experimental and clinical settings, including autoimmune diseases, chronic inflammation, transplantation, and neoplasia (2). IDO1 catalyzes degradation of the essential amino acid tryptophan (TRP) along a pathway that causes TRP starvation and yields several biologically active catabolites, collectively known as kynurenines. Similar to other metabolic enzymes, IDO1 is endowed with a second (“moonlighting”) function, which makes it a signal-transducing molecule owing to the presence of two immunoreceptor tyrosine-based inhibitory motifs (ITIMs) (3, 4). When phosphorylated, those motifs act as docking sites for distinct molecular partners, which can either prolong IDO1’s half-life – thus sustaining immunoregulatory effects – or shorten its half-life, so favoring inflammatory responses (5).

The aryl hydrocarbon receptor (AhR), a ligand-activated transcription factor that mediates dioxin toxicity (6), is of vital importance in the regulation of immune responses. Unbound AhR is sequestered in the cytosol by the Hsp90/XAP2 chaperon complex (7–10). Ligand binding to AhR induces conformational changes that promote nuclear translocation of the receptor. In association with the AhR nuclear translocator (Arnt), AhR modulates the transcription of target genes through AhR-responsive elements (AHREs) (7, 8, 10). A wide array of distinct exogenous and endogenous ligands bind AhR, including indole-containing molecules and TRP metabolites such as l-kynurenine (l-kyn), the upstream metabolite generated via TRP degradation (11, 12). The immunoregulatory effects mediated by AhR have been known to mostly involve its genomic activity, contributing to *Ido1* transcription (13). However, AhR is also involved in a non-genomic pathway, being a component of an atypical ubiquitin ligase complex, which regulates the proteasomal degradation of target proteins (14–16).

### Regulatory proteolysis of IDO1 enzyme

Intracellular proteins are continually “turning over” as they become hydrolyzed to their constituent amino acids and replaced by new synthesis, which absolves to several important homeostatic functions. Cells contain multiple proteolytic systems to carry out the degradation process and complex regulatory mechanisms, to ensure that the continual proteolytic processes are highly selective. In all tissues, the ubiquitin–proteasome system presides over the degradation of the majority of intracellular proteins (17). In dendritic cells (DCs), under inflammatory conditions, IDO1 itself undergoes proteasomal degradation by associating with suppressor of cytokine signaling 3 (SOCS3) through tyrosine phosphorylated ITIMs present in an IDO1 domain distinct from that mediating its enzymatic function (3).

The crystal structure of the enzyme has indeed been solved in its catalytically inactive conformation (18), unveiling the presence of two folding domains, namely, a large domain and a small domain. The former contains the heme-binding site, forming the catalytic cleft of the enzyme, while the latter contains two highly conserved ITIMs, which, once tyrosine phosphorylated, can act as docking sites for the association with different molecule partners (5). Remarkably, the inspection of the crystal structure of the enzyme (pdb code: 2D0T, 2D0U) shows that the phosphorylable ITIM tyrosines are unexposed to the solvent, and thus, poorly compatible with the experimentally observed interaction with SOCS3. Multiple conformational states of enzymes have been reported to play a role in molecular recognition, catalysis, and allosteric modulation (19–21). Compliant to this paradigm, conformational fluctuations of the large and small domains of IDO1 may exist and be triggered by ligand binding and/or by post-translational modifications. Hence, the aforementioned tyrosine phosphorylation of IDO1’s ITIMs may promote a specific conformational state of IDO1, amenable the interaction with SOCS3.

Suppressor of cytokine signaling 3 represents the first IDO1 partner identified so far, capable of binding the ITIM docking sites in the enzyme. As a member of the SOCS protein family, it acts as a feedback inhibitor, blocking JAK/STAT signaling in response to pro-inflammatory cytokines, such as IL-6. SOCS3 possesses a Src homology 2 (SH2) domain, binding phosphotyrosine-containing peptides, and a SOCS box, which recruits an E3 ubiquitin ligase complex and targets several signaling proteins, disparate in nature, for ubiquitination and proteasomal degradation (22, 23). We have previously provided evidence that the SH2 domain in SOCS3, by anchoring phosphorylated IDO1’s ITIMs, brings the enzyme close to the E3 ubiquitin complex that ubiquitinates and targets IDO1 for proteasomal degradation. This mechanism provides an explanation for the observed, inverse relationship between SOCS3 and IDO1 expression in DCs. In fact, in DCs lacking SOCS3, the immunoadjuvant effect of the immunogenic fusion protein CD28-Ig is lost, and the latter behaves much like cytotoxic T lymphocyte-associated antigen 4 (CTLA4)-Ig, which is immune suppressive in nature (24). This has been traced to CD28-Ig’s unique ability to trigger IL-6 and SOCS3 activities, a property unshared by CTLA4-Ig. IL-6-induced SOCS3 is indeed responsible for degrading the protein product of *Ido1*, whose transcriptional activation is mediated by IFN-γ (25). Mutations of the phosphorylable tyrosines in IDO1’s ITIM domains completely abolish the ability of the enzyme to bind SOCS3, thus preventing its targeting for proteasomal degradation (3, 26).

Therefore, the IDO1/SOCS3 association in DCs represents a molecular mechanism whereby IDO1-positive (IDO1^+^) DCs, expressing a tolerogenic phenotype, can turn into immunostimulatory antigen presenting cells (APCs), according to environmental needs (3). According to the variety of pathophysiologic contexts that DCs must face, proteasomal degradation of IDO1 could represent a non-genomic mechanism of modulation of the enzyme in order to promptly turn IDO^+^ DCs into immunogenic IDO-negative DCs under conditions in the local microenvironment that require activation of the immune response. Regulatory proteolysis of IDO1 by the ubiquitin–proteasome system may be a more common event than previously appreciated, and it may involve other pathway besides that of IL-6-induced SOCS3 activation.

### Ligand-dependent E3 ubiquitin ligase activity of AhR

Although AhR has traditionally been defined as a transcription factor involved in adaptive xenobiotic and in environmental pollutant responses – including polycyclic and halogenated aromatic hydrocarbons, such as 2,3,7,8-tetrachorodibenzo-*p*-dioxin (TCDD, “dioxin”) – the direct transcriptional activity of AhR alone does not fully explain its toxicological and physiological effects. Accumulating evidence suggests that AhR exhibits its regulatory functions by “cross-talking” with a variety of signaling pathways, including estrogen (ER) and androgen (AR) receptors (27, 28). In addition to its genomic activity, AhR is also capable of mediating non-genomic effects, by assembling an atypical E3 ubiquitin ligase complex, CUL4B^AhR^ that includes cullin 4B (CUL4B) (14, 28). E3 ubiquitin ligases act in the last step of a sequential reaction, also involving E1 and E2 ligases, and culminating in the ubiquitination of protein substrates.

Similar to the aforementioned ubiquitin ligase activity, mediated by SOCS3 with IDO1 being the target protein, the ubiquitin ligase activity of CUL4B^AhR^ complex has been reported to target several types of protein for proteolysis. Besides the transcriptional regulation of ERs and ARs, ligand-operating AhR has recently been shown to promote proteasomal degradation of the very same receptors, by assembling the CUL4B^AhR^ E3 ubiquitin ligase complex (15). Similarly, ligand-based assembly of the CUL4B^AhR^ E3 ligase complex has been found to promote ubiquitination of β-catenin (16), a transcription factor downstream from the Wnt signaling pathway, leading to proteasomal degradation of β-catenin in colon tumor cell lines. Interestingly, AhR-deficient mice frequently develop colon tumor with abnormal accumulation of β-catenin protein. Inversely, administration of AhR ligands efficiently suppressed colon cancer in an established mouse model of familial adenomatous polyposis. These findings suggest that AhR ligands can be used to successfully prevent intestinal tumors, where increased stabilization and accumulation of β-catenin may be responsible for the initiation of intestinal carcinogenesis. Notably, the substrates of CUL4B^AhR^ ubiquitin ligase complex – ER, AR, and β-catenin – all promote cellular proliferation in their target tissues, suggesting that one putative biological role of the ubiquitin ligase function of AhR could be the antiproliferative activity through degradation of those transcription factors, promoting cell proliferation. This raises the possibility of developing selective AhR ligands in cancer therapy, by promoting ubiquitin ligase function.

Besides a direct role of AhR in assembling an ubiquitin ligase complex, there is evidence that AhR may indirectly promote ubiquitination of target proteins. One such recent mechanism has been described for SOCS2-induced proteasomal degradation of tumor necrosis factor receptor-associated factor (TRAF)6 in a model of *Toxoplasma gondii* infection. In this study, both l-kyn and the lipoxin LXA_4_ were found to induce SOCS2-dependent ubiquitination and proteasomal degradation of TRAF6, hindering pro-inflammatory cytokine expression by DCs. In this case, the mechanism was mediated by the transcriptional activity of AhR, leading to SOCS2 expression that, in turn, promoted TRAF6 polyubiquitination and proteasomal degradation of the adapter proteins (29).

Taken together, the results provide compelling evidence of a prototypic indirect mechanism by which AhR, through AHRE promoter-carrying genes (which include *Socs2* and, interestingly, *Socs3*) can mediate the proteasomal degradation of target proteins. Notably, l-kyn is a TRP metabolite generated by IDO1 in tolerogenic DCs (30), and it acts as endogenous ligand of AhR in promoting IDO1 phosphorylation, leading to TGF-β production (13). Moreover, the interaction of l-kyn with AhR can generate regulatory T cells (12). Overall, these observations shed light on the crucial relationship between TRP metabolism and both the genomic and non-genomic activities of AhR in modulating immune responses.

## Perspective on AhR-Mediated, Non-Genomic Modulation of IDO1

Several experimental models dissected the tight relationship between TRP metabolism and the activity of AhR in modulating the immune response. It is well established that l-kyn, the upstream metabolite generated by TRP-degrading enzymes, acts as an endogenous AhR ligand, leading to generation of regulatory T cells, and participating in immune homeostasis (12, 31). The IDO1–AhR axis has been described in several settings of immune tolerance, including maternal–fetal tolerance (32), immune suppression induced by several human cancers (33), and endotoxin tolerance (13). Therefore, the molecular dissection of the mechanisms that sustain the immunoregulatory IDO1–AhR axis has become a compelling need.

As a transcription factor, AhR promotes IDO1 expression in response to structurally disparate ligands such as l-kyn – in conventional DCs in a model of endotoxin tolerance (13) – and TCDD, during maturation of bone marrow-derived DCs (34), making *Ido1* as an AhR-responsive gene. Moreover, an autocrine signaling loop involving IL-6, STAT3, and AhR was found to sustain the constitutive expression of IDO1 in human cancer cells (33). The genomic modulation of IDO1 by ligand-operating AhR also involves non-canonical mechanisms mediated by kinase activity. TCDD-activated AhR was independently reported to initiate a rapid non-genomic signaling cascade, culminating in the activation of Src and Erk kinases (35–37). Recently, l-kyn–bound AhR was found to promote IDO1 phosphorylation, through Src kinase-mediated activity, which activates the signaling function of IDO1, leading to the *de novo* synthesis of the enzyme via TGF-β production (4, 13). Interestingly, in a model of murine vulvovaginal candidiasis an increased expression of AhR was observed in the vagina of both naïve and infected IDO1-deficient mice, suggesting a further mechanism of mutual transcriptional regulation between IDO1, the source of l-kyn, and its sensor AhR (38).

Besides its transcriptional activity, the non-genomic modality of action of AhR could represent a further mechanism whereby TRP metabolism and AhR cross their pathways. Analogous to sex hormone receptors, regulated by AhR in both transcriptionally and non-genomically fashions, IDO1 could represent another substrate for the ubiquitin ligase activity of CUL4B^AhR^.

Proteasome-mediated degradation of IDO1 has been described in DCs under IL-6-driven pro-inflammatory condition (3). The ubiquitination of the enzyme is mediated by SOCS3 protein that signals the enzyme to the proteasome. The mechanism is particularly active in inflammatory DCs, where SOCS3 is highly expressed and the cells are not required to manifest an immunoregulatory phenotype. Regulatory proteolysis of IDO1 via the ubiquitin–proteasome system may represent a non-genomic means of switching off the enzyme. Similar to SOCS3, the CUL4B^AhR^ complex could promote the ubiquitination of IDO1, targeting it for proteasomal degradation. Physiologically, the non-genomic modulation of IDO1 by ligand-bound AhR could be taken as a typical negative feedback loop of enzyme regulation, where the same trigger (liganded AhR) of its transcriptional expression can also act as a quencher of the protein function, by promoting proteasomal degradation. Such a mechanism would contribute to a fine modulation of IDO1-based immunoregulatory response. The hypothetical view of AhR-driven proteasomal degradation of IDO1 is based on several observations that include the finding that the enzyme is a proteasome substrate (3). Interestingly, both IL-6 and SOCS3 (the trigger of the ubiquitination of IDO1) were independently reported to be induced by activated AhR (33, 39). In addition, SOCS2 – a member of the SOCS family in which SOCS3 likewise belongs – is induced by l-kyn–activated AhR, and it promotes the ubiquitination of the adapter protein TRAF6 (29). Overall, these findings suggest that activated AhR, through its transcriptional activity, is capable of inducing all of the components (i.e., IL-6, SOCS3, and IDO1) of a putative feedback loop, promoting intracellular conditions ensuring IDO1 ubiquitination and proteasomal degradation. In addition, post-translational modifications of substrates, such as phosphorylation, typically serve for recruiting E3 ubiquitin ligases. In fact, tyrosine phosphorylation of the two ITIM motifs in the small domain of IDO1 is needed for anchoring SOCS3 protein that bridges E3 ubiquitin ligase (3). Interestingly, IDO1 phosphorylation may be promoted by AhR activity in conventional DCs in a model of endotoxin tolerance (13).

**Figure 1:**
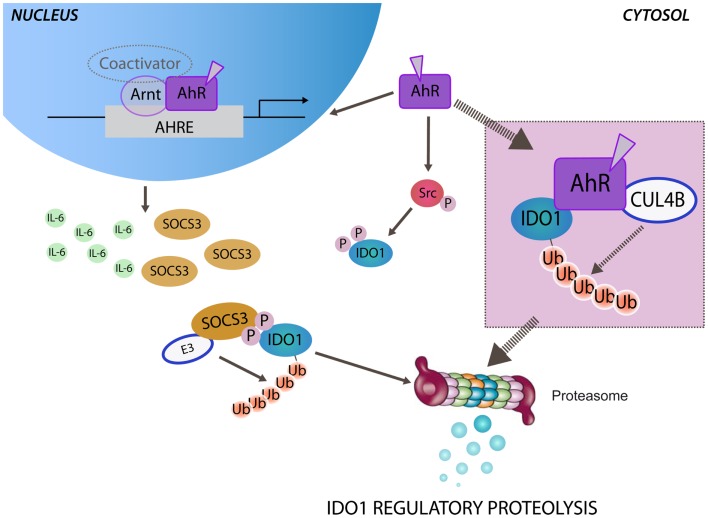
**Aryl hydrocarbon receptor-mediated regulatory proteolysis of IDO1**. Ligand-operating aryl hydrocarbon receptor (AhR) promotes both genomic and non-genomic activity. After nuclear translocation, ligand-bound AhR dimerizes with the AhR nuclear translocator (Arnt) and activates the transcription of target genes through AhR-responsive elements (AHREs). Both IL-6 and SOCS3 are AhR-responsive genes and may be independently induced by the genomic activity of AhR. In the cytosol SOCS3, by anchoring tyrosine phosphorylated IDO1, brings the enzyme close to the E3 ubiquitin complex (E3) that promotes the polyubiquitination and the proteasomal degradation of IDO1. The non-genomic activity of ligand-bound AhR promotes Src kinase-mediated phosphorylation of IDO1, required for anchoring SOCS3. Ligand-bound AhR can assemble an atypical E3 ubiquitin ligase complex, involving cullin 4B (CUL4B), namely, CUL4B^AhR^. A direct association of CUL4B^AhR^ with IDO1 protein has been prospected (inset) in determining the polyubiquitination and the proteasomal degradation of IDO1.

In this hypothetical scenario, AhR would play the canonical role of transcription factor, capable of inducing the stimuli (IL-6 and SOCS3) responsible for ubiquitination of IDO1 enzyme, and would promote – through a non-canonical pathway – the phosphorylation of IDO1 required for anchoring SOCS3. It is also likely that the ubiquitin ligase activity ascribed to AhR might directly act on IDO1 as a substrate, bypassing the “bridging” function mediated by SOCS3 (Figure 1). If so, the direct molecular association of CUL4B^AhR^ with IDO1 enzyme should be characterized. All of the previously described complexes of CUL4B^AhR^ with both ER/AR and β-catenin are localized into the nucleus, as involving the association of ligand-bound AhR with the specific nuclear translocator Arnt. A major question relates to the subcellular location where CUL4B^AhR^ would bind the substrate IDO1. There is no evidence of a nuclear localization of IDO1 enzyme or of a cytosolic localization of CUL4B^AhR^. Thus, one should postulate that AhR assembles an ubiquitin ligase complex that involves different molecular partners, not necessarily requiring the nuclear translocation. In this regard, the C-terminus of Hsp70-interacting protein (CHIP), a quality-controlling ubiquitin ligase, reportedly promotes degradation of AhR. Since both CHIP and unliganded AhR are mainly located in the cytosol, the degradation of AhR through CHIP is likely to occur in the cytosol (40).

A second question relates to the nature of the AhR ligand that could target IDO1 as a substrate for ubiquitination. In view of a fine cross-talk between the TRP metabolism and AhR activity, l-kyn and the downstream TRP metabolites could be good candidates for playing this role in a negative feedback loop, aimed at controlling IDO1 enzymatic activity. As it holds true of ER/AR signaling, in which AhR appears to modulate the receptors both positively and negatively, although l-kyn has been shown to transcriptionally induce IDO1 expression via AhR, later in an inflammatory context, the same molecules generated by IDO1 enzymatic activity could also promote IDO1 ubiquitination and proteasomal degradation.

Thus, l-kyn and its derivatives along the kynurenine pathway, exploiting the genomic and non-genomic modality of action of the receptor AhR, could tightly control IDO1 activity in a sort of negative feedback loop. By construing the ubiquitin ligase activity of AhR as a sensor of environmental stress, as suggested in sex hormone signaling (41), several inflammatory adverse effects of dioxin-type ligands of AhR could be, at least in part, attributed to the accelerated degradation of IDO1 that physiologically prevents overreacting responses. In this regard, the appreciation of exogenous/endogenous ligands that selectively activate the non-genomic pathway of AhR might shed light on the biological role of AhR-based modulation of IDO1.

The involvement of the atypical ubiquitin ligase activity of AhR in the quenching of IDO1 activity may represent an attractive therapeutic perspective. Translated into the clinic, the non-genomic control of IDO1 by activated AhR becomes of great interest in neoplasia. The main strategy currently envisioned to tackle IDO1 clinically is by inhibiting its enzymatic activity. The post-translational modification of the enzyme, promoting its ubiquitination and proteasomal degradation, could represent a valid alternative or a complementary approach to the enzymatic inhibition. In this regard, the purine analog, fludarabine, currently used as a chemotherapeutic agent, has recently been proposed to act as a promoter of proteasome-mediated degradation of IDO1 in tumors (42). Noteworthy, the ubiquitin ligase function of AhR has also been contextualized to the antiproliferative activity resulting from proteasomal degradation of transcription factors (ER/AR, β-catenin) that promote cell proliferation in target tissues. Although IDO1 does not possess a transcriptional activity of its own like other substrates of CUL4B^AhR^, it is noteworthy that, in cancer tissues, IDO1 plays a proliferative action (43–45), and therefore, the putative CUL4B^AhR^-mediated degradation of IDO1 may result in antiproliferative activity.

## Conclusion

Dissecting the molecular mechanism of ubiquitin ligase activity of AhR might lead to a better understanding of the diverse biological effects induced by exogenous/endogenous AhR ligands. Specifically, clarifying this mechanism in relationship to the AhR–IDO1 axis might be of great interest in providing innovative IDO1-based therapeutic targets. AhR-mediated non-genomic modulation of IDO1 might provide druggable targets in cancer therapy, in alternative to or in combination with the already available enzyme inhibitors. Thus, the identification of selective “non-toxic” AhR ligands, activating the non-canonical pathway of the receptor, represents an emerging area of research.

## Conflict of Interest Statement

The authors declare that the research was conducted in the absence of any commercial or financial relationships that could be construed as a potential conflict of interest. The Review Editor Paolo Puccetti declares that, despite being affiliated to the same institution as authors Maria Teresa Pallotta, Francesca Fallarino, Davide Matino, Antonio Macchiarulo and Ciriana Orabona, the review process was handled objectively.
